# What Is the Magical Cavitation Bubble: A Holistic Perspective to Trigger Advanced Bubbles, Nano-Sonocatalysts, and Cellular Sonosensitizers

**DOI:** 10.34133/bmef.0067

**Published:** 2024-09-19

**Authors:** Xiaoge Wu, Fulong Chen, Qi Zhang, Juan Tu

**Affiliations:** ^1^ College of Environmental Science and Engineering, Yangzhou University, Yangzhou 225009, China.; ^2^ Key Laboratory of Modern Acoustics (MOE), Department of Physics, Collaborative Innovation Center of Advanced Microstructure, Nanjing University, Nanjing 210093, China.

## Abstract

Sonodynamic therapy (SDT) has emerged as a novel and highly researched advancement in the medical field. Traditional ultrasound contrast agents and novel bubble-shaped agents are used to stimulate cavitation and enhance SDT efficiency. However, the impact of artificially modified shell structures on the acoustic properties of microbubbles remains to be explored. Alternatively, in the absence of bubble-shaped agents, some clinically available organic sonosensitizers and advanced inorganic materials are also used to enhance the efficacy of SDT. Diagnostic and therapeutic ultrasound can also activate cavitation bubbles, which supply energy to sonosensitive agents, leading to the production of cytotoxic free radicals to achieve therapeutic effects. While inorganic materials often spark controversy in clinical applications, their relatively simple structure enables researchers to gain insight into the mechanism by which SDT produces various free radicals. Some organic–inorganic hybrid sonosensitive systems have also been reported, combining the benefits of inorganic and organic sonosensitive agents. Alternatively, by employing cell surface modification engineering to enable cells to perform functions such as immune escape, drug loading, gas loading, and sonosensitivity, cellular sonosensitizers have also been developed. However, further exploration is needed on the acoustic properties, ability to generate reactive oxygen species (ROS), and potential clinical application of this cellular sonosensitizer. This review offers a comprehensive analysis of vesical microbubbles and nanoscale sonocatalysts, including organic, inorganic, combined organic–inorganic sonosensitizers, and cellular sonosensitizers. This analysis will enhance our understanding of SDT and demonstrate its important potential in transforming medical applications.

## Introduction

Sonodynamic therapy (SDT) is an advanced therapeutic method that employs ultrasound (US) to initiate therapeutic processes. With its increasing prominence in the medical field, SDT has attracted broad interest of researchers and medical practitioners. Although ultrasonic imaging is commonly used in medical diagnosis, the US intensity typically does not exceed 10 mW/cm^2^, and its central frequency usually ranges from 2.5 to 20 MHz, with a velocity in human tissues of 1,540 ± 10 m/s (at 25 °C). However, these settings are not particularly conducive to activating the desired chemical and biological effects required for effective SDT. To prevent potential tissue damage, the exposure time to the human body is typically limited to 30 min [1]. However, it is essential to acknowledge that these constraints mainly relate to US imaging, although historically US’s initial medical application was designed for therapeutic purposes and subsequently found broader use in various scenarios [2].

Recently, SDT has experienced a resurgence due to the integration of sonosensitizers and low-intensity US. Inspired by photodynamic therapy (PDT), SDT has emerged as a novel noninvasive treatment modality for malignant tumors. This approach provides substantial benefits for detecting and treating deep-seated tumors because (a) US penetrates tissue more effectively than light [3]; (b) with the assistance of sonosensitizers, SDT can precisely target the treatment site [4]; (c) and real-time US monitoring is feasible during the treatment. SDT can therefore be used to treat a broader range of deeper and less accessible tumors than traditional PDT [5].

**Fig. 1.**
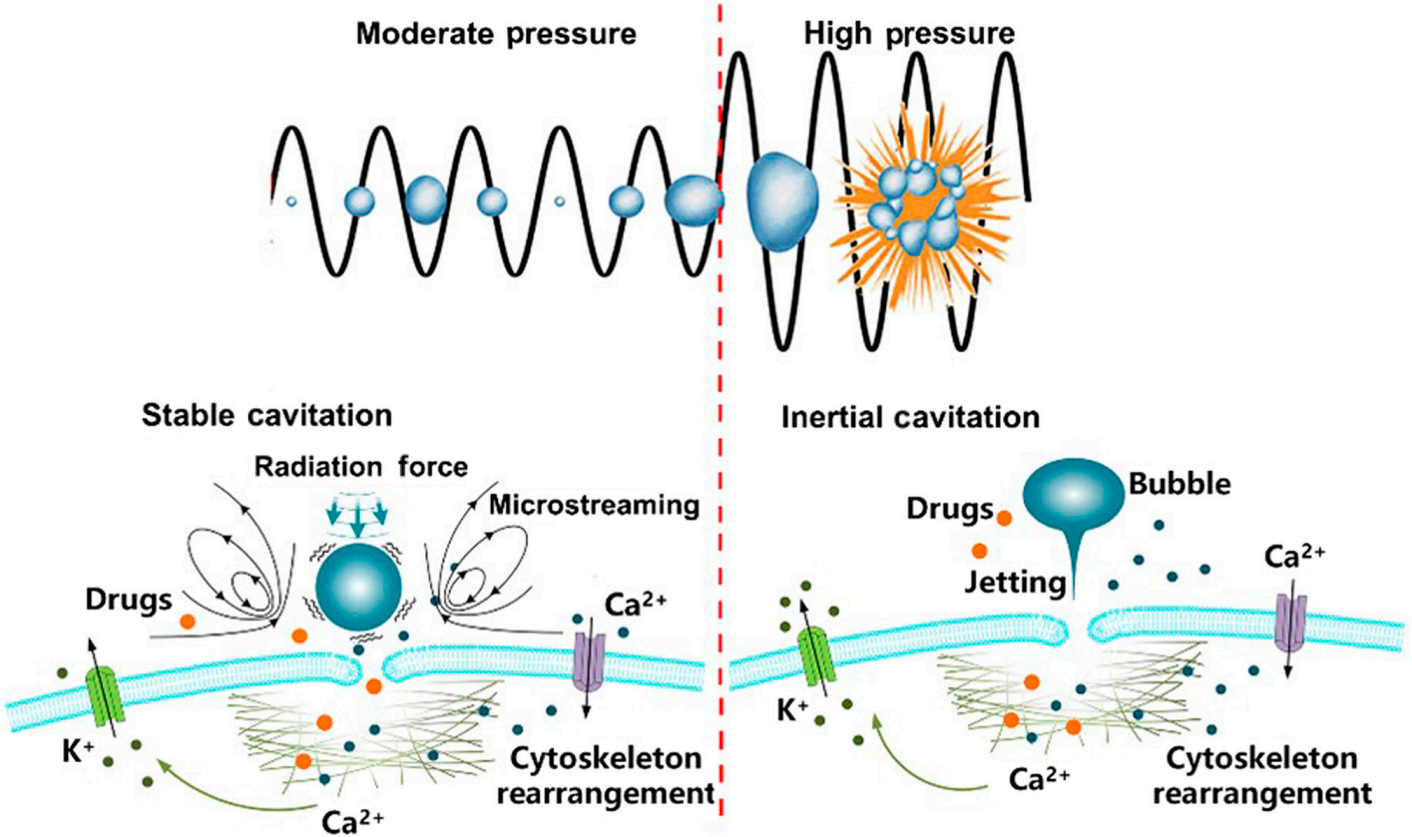
Overview of major biophysical effects occurring during microbubble cavitation. Under moderate pressure, stable cavitation induces volume oscillations that apply pressure and microstreaming-induced shear forces to the adjacent cell membrane. In contrast, inertial cavitation bubbles generate more intense shock waves and microjets. These physical forces can result in cell membrane poration, cytoskeleton rearrangement, and transmembrane iron transport (modified from [7]).

The fundamental mechanism of SDT involves the activation of sonosensitizers by low-intensity US, resulting in the generation of reactive oxygen species (ROS) to treat tumors. Sonosensitizers, therefore, play a significant role in SDT, while the ongoing challenge is to improve the development of more efficient sonosensitizers to advance SDT technology. Sonosensitizers enhance the effects of ultrasonic cavitation, a complex and nonlinear acoustic process in which microbubbles (MBs) in a liquid vibrate under ultrasonic irradiation to act as cavitation nuclei. When the US pressure reaches a certain threshold, the bubbles expand gradually and then collapse quickly. Cavitation processes can be divided into 2 categories: stable cavitation, which generates microstreaming and radiation force, and inertial cavitation characterized by violent bubble collapse resulting in shock wave formation. The combined phenomena of MB vibration, expansion, contraction, and collapse are collectively referred to as acoustic cavitation [6]. During cavitation, the energy accumulated by the cavitation nuclei is rapidly released, leading to extreme physical conditions, including temperatures exceeding 5,000 K, pressures exceeding 100 MPa, and micro-scale jetting within the confined space where cavitation takes place [7]. When a cavitation bubble is in close proximity to a cell membrane, it can efficiently initiate complex biophysical effects, such as membrane sonoporation, Ca^2+^ influx, K^+^ efflux, and cytoskeleton rearrangement (Fig. 1).

**Fig. 2.**
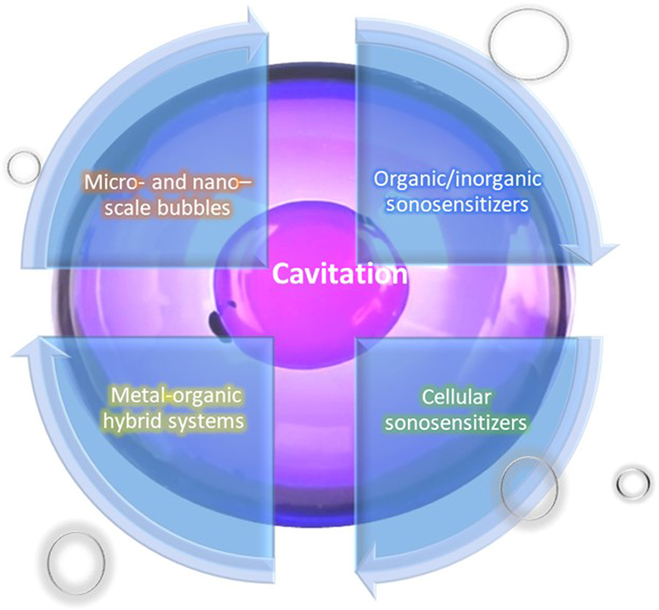
Schematic of advanced sonosensitizers in recent years.

Compared to PDT, the main advantage of SDT is that US can penetrate greater tissue depth noninvasively compared to light. After activation, SDT drugs or “sonosensitizers” will produce ROS, resulting in cytotoxic effects. However, the detailed mechanism of ROS generation is not yet fully understood. This paper reviews typical sonosensitizer including micro- and nanoscale bubbles, alternative sonosensitizers, metal–organic hybrid systems, and cellular sonosensitizers (Fig. 2), and aims to provide a comprehensive discussion and serve as a valuable reference for the improvement of sonosensitizers, ultimately enhancing ROS generation efficacy and facilitating their application in cellular inactivation.

Before examining the advancements in various sonosensitizers, it is important to briefly describe the techniques necessary for their preparation and investigation. Common approaches to synthesizing new materials include hydrothermal synthesis, sol–gel processing, and electrochemical methods. These synthesis methods allow for the creation of nanomaterials in controlled environments, which can improve their size and shape for optimal sonodynamic activity. Techniques such as scanning electron microscopy and transmission electron microscopy provide insights into the morphology and size of newly developed sonosensitizers. Additionally, spectroscopy methods (like ultraviolet–visible and Fourier transform infrared) can assess the chemical properties and functional groups present in the materials. The types and yields of ROS serve as crucial indicators of the performance of sonosensitizers. These parameters are typically assessed using fluorescent probes and electron paramagnetic resonance (EPR) spectroscopy. Furthermore, to confirm the potential therapy effect, in vitro studies using cancer cell lines can aid in understanding how different combinations affect cell viability and apoptosis pathways. Flow cytometry can be employed to measure cell death and assess the efficacy of combination treatment strategies. Initial in vivo studies often utilize mouse models to evaluate the safety and efficacy of sonosensitizers. These models can mimic human cancer conditions, providing valuable data on how treatments perform in a living organism.

## Micro- and Nanoscale Bubbles

Since Gramiak’s groundbreaking report in 1968 that bubbles should be capable of enhancing ultrasonic signals, there has been continuous development in bubbles’ preparation technology and practical applications. Initially used to enhance US image contrast, it was observed that these small gas-filled bubbles produce high-frequency vibrations when stimulated by US. These vibrations can induce circumferential or shear stress on cells, leading to significant biological effects. The latest generation of US MBs can selectively identify the targeted cells or diseased tissues. MBs are generally composed of gas nuclei and stable shell membranes. The coating material of bubbles is composed of lipids, polymers, or proteins. These materials should have good biocompatibility and be relatively safe for intravenous injection. Phospholipids, as the shell membrane of MBs, are more flexible than cross-linked polymer hard shells. Under ultrasonic irradiation, cross-linked polymer hard shells are more susceptible to shrinkage, rupture, bending, or respreading, thereby enhancing the sensitivity of MBs to sound waves. Commercial US contrast agents such as SonoVue all use lipid shell membranes. In addition, the composition of gas nuclei can also affect the stability and functionality of MBs. For example, using oxygen as a filling gas is a smart strategy for applications that require rapid oxygen delivery. However, it is not effective for diagnostic applications that require longer cycle times. Therefore, gases with low solubility, such as perfluorocarbons (PFCs), are often selected for diagnostic applications. Therefore, it is necessary to design and optimize the compositions of MBs.

The vibration, nonlinear acoustic behavior, and free radical generation mechanisms of elastic shell MBs under ultrasonic excitation are fundamental to the biological effects mediated by MBs. The response of MBs to US is significantly influenced by the physicochemical properties of the shell material. Current research on MB preparation mainly focuses on the functional modification of the shell and the associated mechanisms of ROS generation. Studies have shown that stable small-sized MBs are beneficial for drug loading; however, an overly stable shell structure may weaken the acoustic response performance of the MBs, thus limiting their ability to induce cavitation effects and mediate drug delivery [8,9]. Therefore, to reasonably design the shell structure of elastic shell MBs, it is crucial to effectively regulate the acoustic physical and chemical characteristics such as the nonlinear response and cavitation behavior of the MBs. It is urgent to clarify the following key bottleneck issues: (a) improve the kinetic model of encapsulated MBs by considering the effects of shell elasticity and viscosity on the nonlinear changes in the acoustic response of MBs; (b) based on this, explore the optimal range of parameters that can effectively enhance the SDT effect of MBs through experimental studies combining cavitation dosage and ROS yield; and (c) further elucidate the mechanisms of action of the kinetic behavior of ultrasonic sonosensitizers under US [10,11], as well as their chemical and biological effects, by comprehensively considering the correlation between the preparation environmental parameters of MBs, US application parameters, and the efficacy of MB-mediated SDT effects and drug delivery.

Among various applications, drug delivery mediated by US MBs has shown great potential in cancer treatment. MBs can be combined with anticancer drugs or drug carriers to facilitate drug delivery. After US irradiation, MBs in blood vessels undergo cavitation and collapse in tumor tissue. These chemical and mechanical effects will increase vascular permeability. Therefore, anticancer drugs can be specifically delivered at the tumor site to exert therapeutic effects. This method enables highly selective therapeutic drugs to enter tumor cells. Therefore, in theory, US-guided targeted drug delivery can reduce systemic toxicity while ensuring high-dose local administration at the treatment site.

There is a growing number of reports that the cavitation effect generated by US-driven MBs enables the controlled release of drugs or therapeutic genes [12]. For example, a group led by Shen [13] developed a phase change liposome that transitioned from nanoscale liposomes to lipid MBs and back through US stimulation. Simultaneously, this liposome could switch from a negative to a positive electrical potential, triggered by the acidic microenvironment of a tumor. This unique property facilitated deeper penetration into solid tumors and enhanced drug activity, providing a novel drug delivery system with excellent biocompatibility and controlled drug release using functional MBs. Moreover, Yan’s group successfully combined customized liposomes with neutrophils to create oxygen-carrying sonosensitizers for cancer diagnosis and treatment. This innovative approach imparts new characteristics to cells for effective medical treatment [14].

**Fig. 3.**
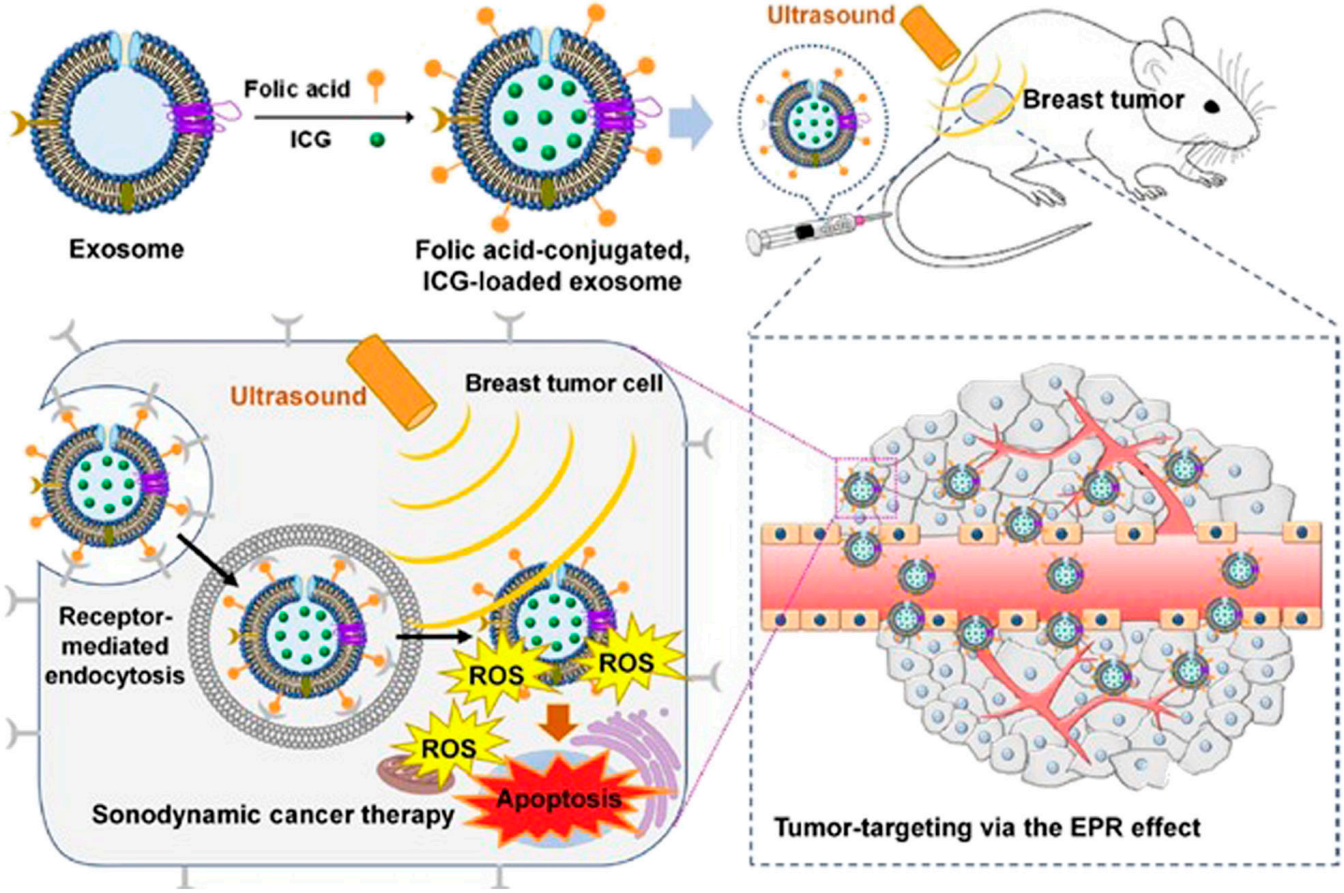
Structures of a modified exosome and the related behaviors in a sound field. Reproduced with permission [15]. Copyright 2021, American Chemical Society.

Besides lipid vesicles, some natural vesicle structures are also used as contrast agents. For example, exosomes are extracellular vesicles with high biocompatibility, long blood circulation half-life, and low immunogenicity, which can effectively transport various hydrophobic and hydrophilic drugs. They have also been used as sonosensitive bubbles for drug delivery. In Fig. 3, a nanosystem for surface-engineered extracellular vesicle delivery of sonosensitive agents is explained in [11]. Using folic acid surface modification of exosomes derived from HEK-293T cells, through receptor mediation, the endocytosis of cancer cells was enhanced by the modified exosomes, leading to higher tumor-targeting ability. Then, the sonosensitive agent indocyanine green (ICG) was further loaded. Thus, this active tumor-targeted extracellular vesicle can serve as an effective nano-sonosensitizer for safe and targeted cancer treatment [15].

In this way, micro- and nano–scale bubbles can effectively serve as carriers for drugs, and their rupture actively by US exposure allows for controllable drug release. Table 1 presents some of the representative works in this field. In describing the modification and application of MBs, we have also included ultrasonic parameters in the table, offering valuable insights into the impact of ultrasonic parameters and physicochemical properties of MBs on the efficacy of SDT. It should be pointed out that the gap between endothelial cells in the tumor or inflammatory site can reach about 780 nm. Therefore, nanoscale contrast agents can penetrate the tissue through the vascular gap at the lesion site. When nanoparticles carry drugs and genes, they can maximize drug enrichment within the solid tumor, thus achieving good therapeutic effects [16]. Compared with micrometer-sized MBs, nanoscale US contrast agents can enable extravascular imaging of the lesion site through the endothelial gap, which indicates that nanoscale contrast agents also have significant US enhancement capability with a sufficiently high concentration, although their size is relatively small [17]. At present, further exploration and research are required to maintain the stability and strong sonosensitivity of nanoscale US contrast agents in vivo. Yeh and colleagues [18] reported a sonosensitizer—carbon dots (C-dots)—which is used to assemble MBs (C-dots MBs) with a gas core. The C-dots are directly integrated into the shell of the MBs, allowing them to effectively absorb the energy from inertial cavitation and transfer it to ROS. This paves the way for the design of novel multifunctional sonosensitizers for SDT in tumor treatment.

**Table 1.** Microbubbles and nanocapsules for SDT

Sonosensitizers	Application	US conditions	Reference
Exosome-based sonosensitizers	Breast cancer cells	1 MHz, 0.3 W cm^−2^, 2 min	[15]
Rose bengal microbubbles with nanoparticles	Colon cancer cells	1 MHz, 1.5 W cm^−2^, 50% duty cycle, 900 s	[67]
Oxygen-sufficient lipid nanobubbles	Liver cancer cells	1 MHz, 3.0 W cm^−2^, 120 s	[68]
Perfluoropentane liposome loaded with IR780	Breast cancer cells	650 kHz, 0.8, 1.6, 2.4, 3.2, 4.0 W cm^−2^, 3 min	[69]
Nanoscale N_2_O microbubbles	Breast cancer cells	0.94 MHz, 125 W, 5 s	[70]
Iridium complex-loaded perfluoropropane nanobubbles	Breast cancer cells	1 MHz, 1.7 W cm^−2^, 1 min	[71]
Triphenylphosphonium hollow nanocapsules	Cervical cancer cells	1 MHz, 2.0 W cm^−2^, 10% duty cycle, 10 min	[72]
Manganese protoporphyrin liposomes	Breast cancer cells	1 MHz, 2.0 W cm^−2^, 50% duty cycle, 3 min	[73]

The development of novel micro- and/or nanobubble for US contrast agents with high stability and drug loading holds significant scientific and medical value. However, creating stable small-sized MBs is a challenging endeavor. As shown in Table 1, the duration of US exposure to MBs and nanocapsules is relatively short, because the bubbles’ sonosensitivity would be easily lost with their ruptures induced by long-term sonication. In addition, the efficiency of lipid vesicles in catalyzing the production of ultrasonic free radicals is very low. Therefore, researchers are committed to developing sonosensitive agents with good biocompatibility and high sonosensitive efficiency. Staying true to this concept, researchers are actively engaged in the development of organic and inorganic sonosensitizers. In the following sections, we will explore their preparation, sonosensitivity mechanisms, and biological effects.

**Fig. 4.**
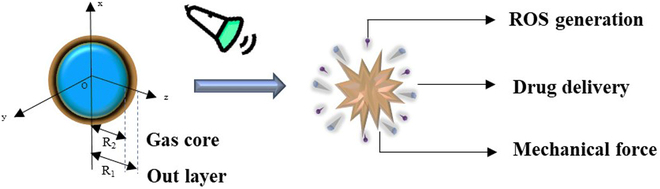
A schematic diagram of the proposed response of a multifunctional bubble to US. It is an important theoretical basis for cavitation-mediated biological effects.

In fact, studying the nonlinear dynamic behavior of MBs under US is an important theoretical basis for the biological effect mediated by MBs. A typical bubble for ultrasonic application is that the gas core is wrapped in an outer layer of material, and the response of MBs to US is greatly affected by the physical and chemical properties of the shell material (Fig. 4). At present, the research focuses on the functional modification of MBs and related ROS production mechanisms (section “SDT Without Micro- and Nanoscale Bubbles”). There is insufficient research on the mechanical force-mediated biological effects induced by drug delivery and ultrasonic stimulation of MBs (Fig. 4). However, preparing stable small-sized bubbles is not an easy task, and an overly stable shell structure weakens the acoustic response performance of MBs, limiting US-controlled explosive controlled-release drugs. How to design the shell structure of MBs to effectively regulate the nonlinear response and acoustic cavitation behavior of MBs needs to be explored. A perfect bubble model should consider the nonlinear changes in shell elasticity and viscosity at the same time. Moreover, monitoring methods combining cavitation dose and ROS production should be constructed to verify the validity of the encapsulated MB dynamic model. However, due to limitations of preparation technology, delivery efficiency, and acoustic parameters of MBs, the exact mechanism of the MB dynamics as well as chemical and biological effects of the acoustic sensitizer MB system are still unclear.

## SDT Without Micro- and Nanoscale Bubbles

Excellent sonosensitizers should possess both thermodynamic and kinetic factors to produce high yields of ROS. The narrow bandgap of sonosensitizers is a beneficial thermodynamic factor. Therefore, the generation of electron-hole (e^−^-h^+^) pairs in low-intensity US settings only requires minimal energy. Studies have shown that the prolonged excited state lifetime of sonosensitizers is a favorable kinetic factor, as long-lasting charge carriers are likely to participate in catalytic reactions before the recombination of e^−^-h^+^ pairs [19]. In this regard, sonosensitizers should have narrow bandgaps and extended carrier lifetimes to effectively excite, separate, and catalyze charge carrier, for the following ROS reactions.

**Fig. 5.**
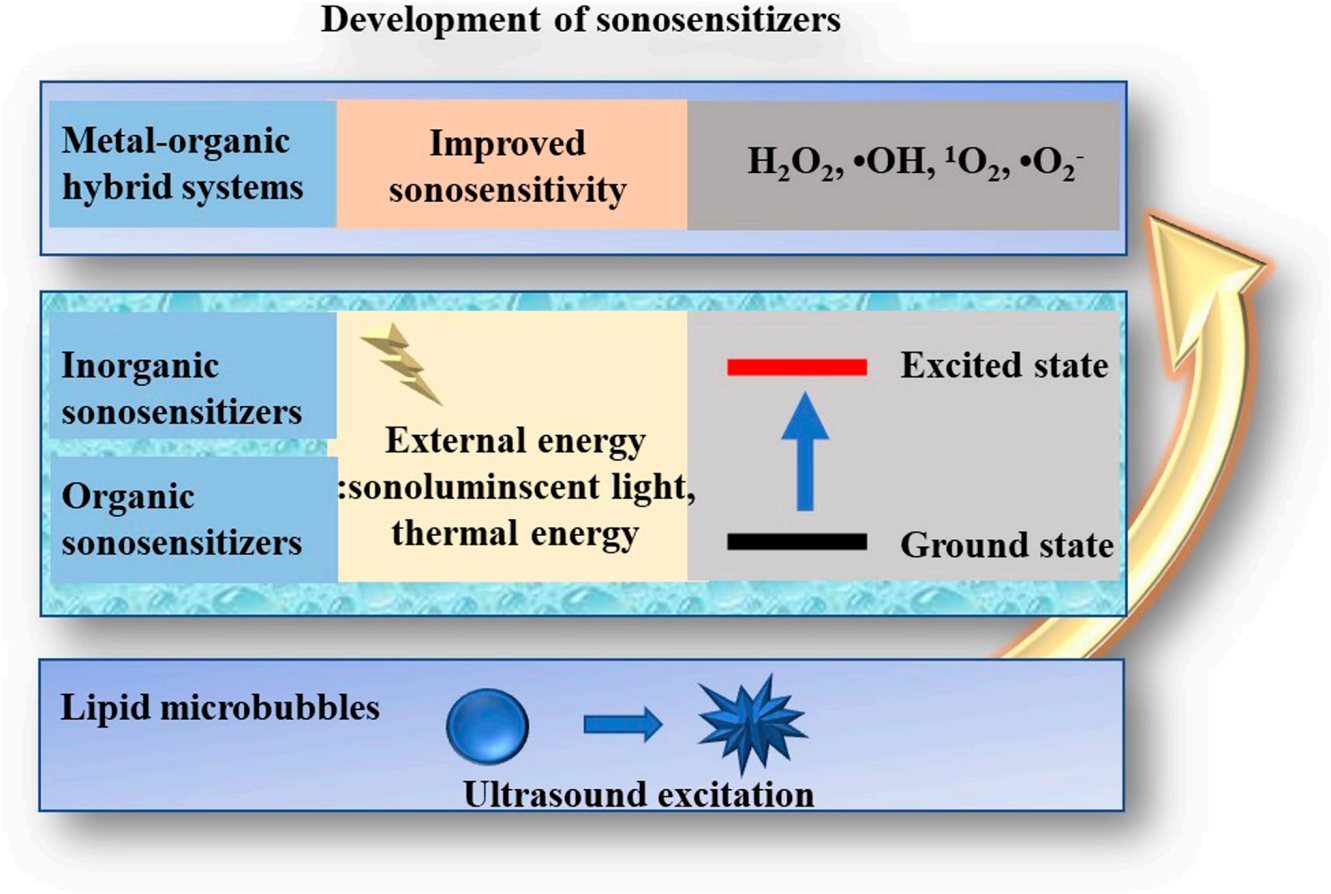
Overview of sonosensitizer structures, activation mechanisms, and applications.

Nowadays, SDT utilizes acoustic cavitation to generate ROS, which can attack tumor cells, bacteria, and other harmful microbial cells. Several sonosensitizers have gained substantial attention among the reported options, including lipid MBs, organic sonosensitizers (e.g., porphyrin derivatives and phthalocyanines), inorganic sonosensitizers (e.g., titanium oxide), and metal–organic sonosensitizers (e.g., Mn-methylphenylporphyrin) (Fig. 5). Although progress of sonosensitizers has been made in the emerging field of SDT, the low production of ROS produced by sonosensitizers is one of the main limitations of clinical translation. Thus, the sensitivity of the selected sonosensitizer is a key factor in determining the effectiveness of SDT.

### Organic sonosensitizers

Traditional organic sonosensitizers are mainly composed of natural products like porphyrin, chlorophyll, hypocrellin, turmeric, organic dyes (e.g., cyanine and rose red), or organic nanoscale particles. This is due to their unique characteristics, which include a wide range of π-electron conjugate systems and multifunctional photoelectric and catalytic properties [20–23]. However, organic sonosensitizers often show limited stability under US irradiation, leading to poor SDT efficiency. Currently, clinically available sonosensitizers commonly suffer from issues related to oxygen dependence and skin phototoxicity. Hence, the current research focus has shifted toward the development of novel and more efficient organic sonosensitizers. Ni and colleagues [24] synthesized nanoscale polyethylene glycol (PEG) droplets containing an amino sonosensitizer, tetrakis (4-carboxyphenyl) porphyrin (TCPP), and divalent copper ions. The copper ions are chelated inside TCPP molecules. The material has been shown to be activated by glutathione overexpressed in the tumor microenvironment (TME), leading to selective SDT. In addition, the inclusion of PEG imparts prolonged blood circulation time to the nanoparticles, promoting the accumulation of nanoparticles around tumors, thereby further enhancing the treatment effectiveness. In order to ensure safety and improve clinical transformation potential, researchers have employed hydrophilic–hydrophobic self-assembly technology to assemble the hydrophobic organic dye chlorin e6 (Ce6) with the broad-spectrum anticancer drug paclitaxel (PTX) and the hydrophilic organic dye IR783 into a nanoscale photosensitizer designated as Ce6-PTX@IR783. Integrating multiple molecules into a nanoscale system delivers sonochemical effects activated by US along with photoacoustic imaging. The Ce6 enhances the SDT, PTX provides the targeted chemotherapy, and IR783 contributes to both enhanced tumor-specific accumulation and effective photoacoustic imaging. The small particle size (70 nm) of Ce6- PTX@IR783 promotes its accumulation in tumors by enhancing permeability and retention [25]. Another innovative approach reported by Zhang’s group [26] involves the use of a simple precipitation method to prepare nanoparticles with aggregation-induced luminescence as sonosensitizers for enhanced SDT. By combining the advantages of SDT and PDT, this approach aims to further enhance the therapeutic effect. Furthermore, Shen [27] has designed a covalent organic polymer that exhibits good physiological stability and sonosensitization, effectively triggering cancer cell death under ultrasonic irradiation.

The researchers designed and synthesized triphenylthiophene compounds doped with triarylboron, which can efficiently generate hydroxyl radicals (•OH) when exposed to US. The researchers propose a potential mechanism in which the boron-containing group within the sonosensitizer’s small molecule serves as an electron acceptor. This electron acceptor property facilitates the transfer of electrons from the hydroxyl group under US, promoting the generation of hydroxyl radicals. Preliminary theoretical calculations confirm that boron-containing molecules are more favorable for electron transfer reactions compared to boron-free molecules. Cell experiments demonstrate that boron heterobenzothiophene can effectively eradicate tumor cells under sonication, demonstrating exceptional sonochemical efficiency. Furthermore, the investigation of ROS under illumination reveals that boron heterobenzothiophene distinguishes itself from traditional organic sonosensitizers and successfully addresses the issue of skin phototoxicity at therapeutic concentrations [28]. Li et al. [29] synthesized BODIPY dyes based on the design principles of photosensitizers, and the results indicated that a large conjugated π-electronic system with a coupled structure may be beneficial for the ultrasonic cavitation effects of BODIPY.

The development of organic sonosensitizers has shed new light on the sonochemistry involved. Although the progress in organic sonosensitizers holds great promise, there is a need for further improvement in their stability. Additionally, it is worth noting that many organic sonosensitizers also function as photosensitizers, and their phototoxicity remains a concern in the context of tumor treatment applications.

### Covalent organic frameworks

Apart from assembling various traditional organic sonosensitizers and anticancer drugs or synthesizing new organic sonosensitizers, researchers have also explored the use of covalent organic frameworks (COFs), a novel material, in SDT research. For example, hollow COF (HCOF) is an ideal carrier for drug delivery because of its unique structure and abundant internal cavities. Researchers have developed a 2-step controlled synthesis method for HCOFs, involving solvent evaporation and oxidation of imine bonds. Based on this, the researchers skillfully constructed HCOF libraries with different nanostructures, including bowl-, egg yolk shell-, capsule-, and flower-shaped shapes. Because of its large cavity, HCOFs can serve as an ideal carrier for drug delivery. Thus, HCOF was used as a nanocarrier, loaded with 5 small molecular acoustic sensitizers: doxorubicin hydrochloride (DOX), curcumin, Bengal rose red (RB), ICG, and Ce6. The utilization of HCOF loaded with Ce6 is beneficial for separating electrons and holes, facilitated by its narrow bandgap, resulting in enhanced in vivo SDT [30].

**Fig. 6.**
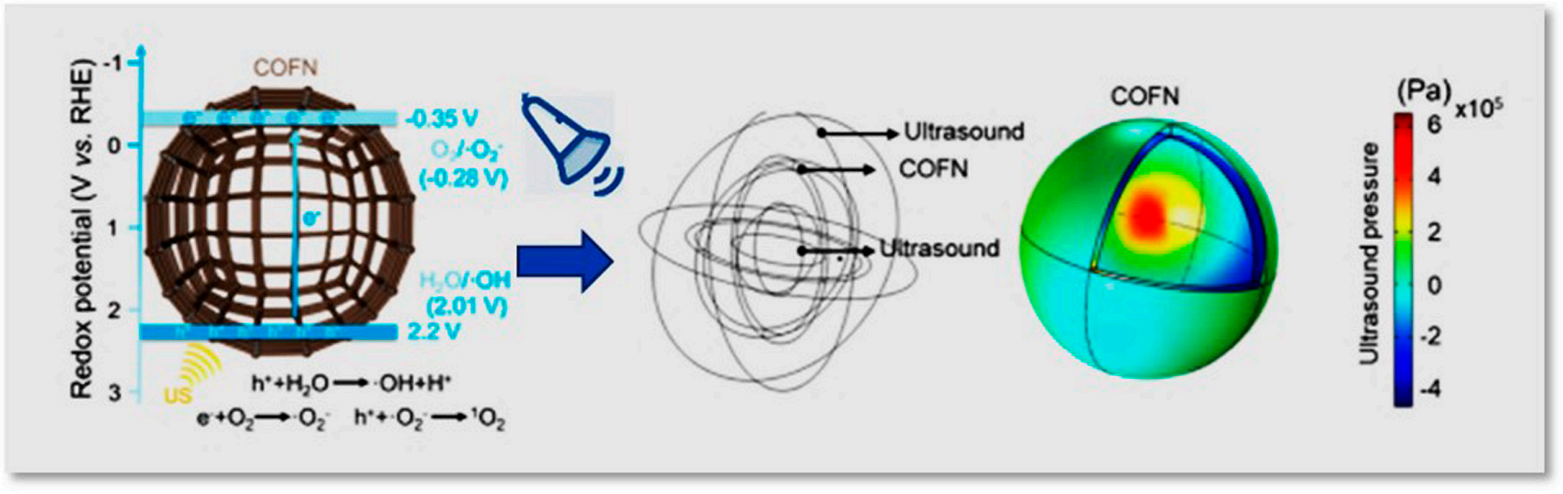
Mechanism of the enhanced sonodynamic performance in COFN (modified from [31]). Copyright 2023, ACS Publications.

A COF nanocarrier (COFN) is a novel platform with great potentials, which is used as an efficient sonosensitizer to promote the research of free radical generation and cancer treatment. COFN is created through the condensation polymerization of porphyrin derivatives via imine bonds. It possesses numerous advantages, including a hollow, porous structure, π-electron conjugate structure, and efficient electron transport behavior, all of which contribute to enhancing the performance of SDT (Fig. 6). COFN has a nanocage structure with a diameter of about 170 nm and a shell thickness of about 36 nm. In comparison to porphyrin small molecules, COFN exhibits a significantly reduced absorption peak at around 430 nm, effectively mitigating the phototoxicity associated with porphyrin small molecules. The porous hollow structure and high specific surface area of COFN facilitate the exchange of reactants, gas adsorption, and gas exchange during redox reaction, thus enhancing the ultrasonic cavitation effect. The results of cell and animal experiments show that, combined with US exposure, COFN has a significant antitumor effect on mouse breast cancer cells and mouse subcutaneous tumor [31].

Li et al. [32] reported that the polymerization reaction between the 2 monomers, 2,2′-diaminodiphenyl disulfide and 2,4,6-trihydroxybenzaldehyde, quickly results in the formation of porous organic polymer nanoparticles within a short timeframe. These nanoparticles effectively encapsulate the antitumor drug mitoxantrone, yielding drug-loaded nanoparticles. To enhance the sonodynamic effects of the material, the sonosensitizer hematoporphyrin monomethyl ether (HMME) is loaded onto the surface of the nanoparticles. Subsequently, the nanoparticles are coated with homologous tumor cell membranes to improve their hydrophilicity and prolong their circulation time in vivo. ROS were generated using ultrasonic irradiation, which induce oxidative stress and activate the type II immunogenic cell death pathway.

### Inorganic sonosensitizers

In the current controversial situation of SDT mechanisms, the study of mechanisms in complex SDT systems is challenging. Developing efficient inorganic sonosensitizers to generate ROS is of great significance for exploring the mechanism of SDT [33]. Alternatively, inorganic sonosensitizers possess high stability, making them attractive candidates for SDT. Nonetheless, they often exhibit low sonosensitization efficiency, and concerns have emerged regarding their extended retention within the body, requiring further research. This area is currently under active investigation. These research studies mainly focus on the production of many free radicals under the activation of US by regulating the physical and chemical structure of sonosensitizers. These nanoparticles have narrow bandgaps and can efficiently separate electron and hole pairs under US activation, generating ROS. SDT with inorganic sonosensitizers has been conducted using both high-frequency (>1 MHz) and low-frequency (40 kHz) US (Table 2), resulting in the production of ROS such as •OH, ^1^O_2_, and •O_2_^−^.

**Table 2.** Inorganic sonosensitizers for SDT

Sonosensitizers	ROS	Application	US conditions	Reference
Porous lanthanum-doped manganese oxide	•OH, ^1^O_2_	Breast cancer cells	20 kHz, 3 W cm^−2^, 30 s	[74]
Piezoelectric Bi_2_MoO_6_ nanoribbons	•OH, ^1^O_2_, •O_2_^−^	Uterine and cervical cancer cells	40 kHz, 3 W cm^-2^, 50% duty cycle, 5 min	[75]
Fe-doped vanadium disulfide nanosheets	•OH, ^1^O_2_	Breast cancer cells	40 kHz, 6.5 W cm^−2^, 50% duty cycle, 10 min	[76]
Vanadium tetrasulfide (VS_4_) as a narrow bandgap sonosensitizer	•OH, ^1^O_2_, H_2_O_2_	Breast cancer cells	1 MHz, 1.5 W cm^−2^, 50% duty cycle, 5 min	[77]
Graphene quantum dots with pyrrole N and pyridine N	•OH, ^1^O_2_	Breast cancer cells	1 MHz, 2.5 W cm^−2^, 50% duty cycle, 1 min	[47]
Titanium carbide nanosheets with defects	^1^O_2_	Breast cancer cells	40 kHz, 3 W cm^−2^, 1 min per cycle, 15 cycles	[78]
Platinum crosslinked p–n carbon dot@TiO_2−x_ junctions	•OH, ^1^O_2_,	Osteosarcoma cells	50 kHz, 2.0 W cm^−2^, 5 min	[79]
TiO_2_ nanosheets with the Au nanocrystal-decorated edges	^1^O_2_	Breast cancer cells	1 MHz, 1.5 W cm^−2^, 5 min	[80]
Ultrasmall iron-doped titanium oxide nanodots	^1^O_2_	Breast cancer cells	40 kHz, 3 W cm^−2^, 33% duty cycle, 15 min	[34]
Oxygen-deficient BiOCl	^1^O_2_, •O_2_^−^	Breast cancer cells	1 MHz, 1.5 W cm^−2^, 50% duty cycle, 5 min	[81]
Au-TiO_2_ nanocomposite	^1^O_2_	Squamous carcinoma cells	1.5 MHz, 30 W, 2 min	[82]
Few-layer black phosphorus nanosheets	^1^O_2_, O_2_^−^	Breast cancer cells	1 MHz, 2.5 W cm^−2^, 10 min	[83]
MnO*_x_*/TiO_2_-graphene oxide	^1^O_2_	Breast cancer cells	1 MHz, 1.0 W cm^−2^, 50% duty cycle, 10 min	[84]
Single-crystal mesoporous TiO_2_	•O_2_^−^, •OH, HO_2_•	Liver cancer cells	0.8 MHz, 1.5 W cm^−2^, 50% duty cycle, 1 min	[85]
TiO_1+*x*_	^1^O_2_, •OH	Breast cancer cells	40 kHz, 3.0 W cm^−2^, 50% duty cycle, 5 min	[35]
Ultrasmall oxygen-deficient bimetallic MnWO*_x_*	^1^O_2_, •OH	Breast cancer cells	40 kHz, 3.0 W cm^−2^, 50% duty cycle, 5 min	[86]
Silicon nanowires decorated with platinum	^1^O_2_, •OH	Breast cancer cells	40 kHz, 3.0 W cm^−2^, 50% duty cycle, 5 min	[87]
Oxygen-deficient ZrO_2−x_	•OH	Breast cancer cells	1 MHz, 0.5 W cm^−2^, 50% duty cycle, 2 min	[88]

One of the research focuses is how to effectively improve the efficiency of sonodynamic response of sonosensitizers. While it is commonly accepted that nanomaterials can offer cavitation nucleation sites, lower cavitation thresholds, and facilitate energy transfer, thereby enhancing ultrasonic chemical, thermal, and biological effects, the exact mechanism of the sonodynamic phenomenon requires further exploration. In this paper, we divide the inorganic sonosensitizers into 5 categories: semiconductors, Fenton- and enzyme-like catalysts, hollow catalysts, low-dimensional materials, and inorganic sonosensitizers fabricated from organic–inorganic materials. We then analyze the mechanisms underlying these sonosensitizers.

#### Semiconductors

Sonosensitizers with a gas core and semiconducting properties generate carriers that are separated and diffuse to the surface of the sonosensitizer. Electrons from the conduction band are captured by O_2_, resulting in the generation of abundant p–n •O^2−^ ions, while holes from the valence band can generate •OH when combined with OH^−^ in water. Electron induction can also convert some of the •O^2−^ to •OH. The acoustic effects of these sonosensitizers can be significantly enhanced by controlling the structure and chemical composition of the semiconductor material, such as by forming ultrathin sheets or inducing chemical defects.

Liu and colleagues [34] have found that nanoscale metal oxides can be efficiently prepared by liquid exfoliation if the exfoliation reagent is well matched with the surface energy of the metal oxide. Notably, TiH_1.924_ nanodots can produce ROS under US stimulation, showing an efficient sonosensitization effect. Interestingly, TiH_1.924_ nanodots exhibit robust near-infrared absorption, allowing their gentle photothermal effect to improve blood flow within tumors and increases their oxygen content, thereby synergistically enhances PDT and SDT. Cheng’s group [35] has successfully synthesized ultrafine titanium oxide (TiO_1+*x*_) nanorods, which exhibited more efficient reactive oxygen generation than most TiO_2_ sonosensitizers, potentially due to their oxygen defect structure, which restricts the recombination of electron-hole pairs excited by US. Also, TiO_1+*x*_ nanorods (NRs) have peroxidase-like activity and can catalyze excess hydrogen peroxide in tumors to produce hydroxyl radicals, leading to chemokinetic therapy (CDT). Moreover, after PEG modification, TiO_1+*x*_ NRs did not show any obvious long-term toxicity. Therefore, PEG-TiO_1+*x*_ NRs could be considered as a promising candidate for use as a sonosensitizer.

Furthermore, Zhang and colleagues [36] have fabricated a gadolinium-doped zinc oxide semiconductor rich in defect sites and used it for efficient SDT of deep tumors. The presence of numerous oxygen defect sites can facilitate the separation of electrons and holes within a sonosensitizer, greatly enhancing its therapeutic effectiveness. Due to the high concentration of oxygen defect sites in the sonosensitizer, it can adsorb water and oxygen molecules, leading to a substantial improvement in ROS generation. This is very useful in the treatment of deep breast cancer using ZnO nanomaterials with rich defect sites. This research not only broadens the medical applications of nanoscale semiconducting materials but also provides valuable insights into the underlying therapeutic mechanism by modulating the physical and chemical properties of these semiconductors. This opens up new possibilities for the use of semiconductors in SDT.

Bismuth (Bi)-based biomaterials have gained extensive research attention in the biomedical field due to their excellent biocompatibility and unique physicochemical properties. Bi-based nanomaterials are considered wide bandgap semiconductors capable of injecting excited electrons into the conduction band of the Bi-based nano-semiconductors under ultrasonic stimulation, enhancing their reducing capacity and consequently generating more superoxide anion radicals. Li and colleagues [37] reported a porphyrin-sensitized Bi-based nano-semiconductor as a sonosensitizer for SDT, which produces ROS and synergistically depletes glutathione to enhance the therapeutic efficacy of tumor sonodynamic treatment. By closely contacting metallic Bi and oxygen-deficient Bi_2_O_3_, a homogeneous Schottky heterojunction can form within the nanostructure. The Bi-based sonosensitizers developed using this strategy exhibit significant efficiency in generating singlet oxygen (^1^O_2_), superoxide (•O^2−^), and hydroxyl radicals (•OH), making them suitable for combined tumor treatment [38]. Moreover, the Bi-based ternary heterojunction semiconductor Bi@BiO_2−x_@Bi_2_S_3_ improves the yield of US-triggered catalytic active oxygen generation through charge separation engineering. This material exacerbates and amplifies oxidative stress damage to cells by regulating glutathione depletion and thermal injury. This charge separation regulation method not only advances the application of Bi-based materials as sonosensitizers in sonodynamic tumor therapy but also provides a thermal injury-assisted strategy, offering new ideas and methods for the design of novel sonosensitizers and improving the efficacy of SDT [39]. Additionally, ultrafine lithographically patterned Bi vanadate (BiVO_4_) nanorods have achieved in situ self-supplying oxygen and active ROS for hypoxic tumor treatment. The lithography method enhances charge separation by inducing oxygen-rich vacancies on the surface of BiVO_4_, thereby increasing the efficiency of ROS and O_2_ generation [40].

#### Fenton- and enzyme-like sonosensitizers

Sonosensitizers with good Fenton catalytic activity can enhance the yield of free radicals. For example, titanium oxide can catalyze the production of hydroxyl radicals from H_2_O_2_ under US irradiation [35]. In this regard, the cycling of elemental iron species in iron-doped titanium oxide nanodots has been shown to possess a Fenton catalytic function that can catalyze H_2_O_2_ to produce ROS [34].

US irradiation can also induce MnCO_3_. Nanoparticles (NPs) can produce hydroxyl radical (•OH) and singlet oxygen (^1^O_2_). Furthermore, in an acidic microenvironment, MnCO_3_ nanoparticles can release CO_2_ and Mn^2+^. The resulting CO_2_ bubbles can enhance cavitation, leading to the irreversible necrosis of cancer cells. The gas also improves US imaging. Additionally, Mn^2+^ can induce cancer cell apoptosis by disrupting mitochondrial function. In vivo experiments have shown that MnCO_3_ nanoparticles alone can achieve a tumor inhibition rate of about 50%. With US stimulation, this rate can reach 90% [33]. The SDT synergistic anticancer treatment proposed by this research not only broadens the application scope of sonosensitizers but also provides new ideas and insights for the advancement of nanoscale diagnostics.

Recently, a glassy material known as IrTe_2_ (G-IrTe_2_) was developed for SDT/CDT/PTT (photothermal) synergistic tumor therapy. G-IrTe_2_ can produce ^1^O_2_ when exposed to US, achieving SDT. G-IrTe_2_, exhibiting cat-like activity, can produce O_2_ by consuming overexpressed H_2_O_2_ in TME. This alleviates TME hypoxia and provides raw materials for SDT, enhancing ^1^O_2_ production. Theoretical calculations suggest that the weak Ir–Te bond of amorphous G-IrTe_2_ is more susceptible to break and generate free electrons under the stimulation of US. G-IrTe_2_ is better suited for activating O_2_ and promoting its involvement in the production of ^1^O_2_, simplifying the realization of SDT. In addition, G-IrTe_2_, which possesses POD-like properties, can facilitate outstanding CDT, and •OH diversifies the range of ROS. In addition, the rapid increase of ROS in tumor cells leads to mitochondrial dysfunction and significant down-regulation of heat shock protein (HSP) expression, resulting in mild PTT with effective G-IrTe_2_. In short, G-IrTe_2_ can deliver a favorable therapeutic outcome through the combination of SDT, CDT, and ROS-activated mild PTT. This work provides a model for the treatment of malignant tumors using enzyme-like acoustic sensitizers [41]. Dinuclear iridium (III) induces cell death upon ultrasonic stimulation, and these dying cells can release pro-inflammatory factors, leading to the activation of CD8^+^ T cells and achieving immune SDT [42].

#### Mesoporous nanoparticles

Nanoparticles with a mesoporous structure can serve as a sonosensitizer with drug loading functionality. The mesoporous structure promotes the separation of electrons and holes, resulting in enhanced yields of free radicals. For example, Lin and colleagues [43] prepared targeted and biodegradable nanoscale sonosensitizers based on hollow, mesoporous organosilicon NPs. These novel nanosensitizers effectively carried the chemotherapy drug doxorubicin (DOX), allowing controlled release within hepatocellular carcinoma (HCC) cells through US stimulation. As a result, the chemotherapeutic effect of the drug was significantly enhanced. Lin and colleagues [44] have reported on the testing of hydrogenated hollow Pt-TiO_2_ nanoparticles for enhancing the efficacy of SDT. The hollow mesoporous TiO_2_ nanoparticles were initially prepared, followed by the deposition of a Pt layer onto the TiO_2_ surface using a straightforward and controlled vacuum metal sputtering deposition technique. With further hydrogenation, Pt-TiO_2_ nanoparticles formed an anoxic layer on the surface of TiO_2_ and the deposited Pt layer to recombine into heterogeneous Pt nanoparticles. With an increased hydrogenation temperature or time, nanoparticles migrate to cover the entire TiO_2_ surface. The disordered shell and the deposited Pt nanoparticles promote the separation of electrons and holes from the energy band structure of TiO_2_ under US radiation, leading to an enhanced therapeutic effect [44].

#### Low-dimensional materials

Certain “2-dimensional” (2D) materials also show good sonosensitivity. For example, Tao’s group synthesized tin nanosheets by liquid-phase ultrasonic exfoliation and evaluated their potential as sonosensitizers. These nanosheets efficiently generated ^1^O_2_ and •OH when subjected to US activation, resulting in the effective eradication of tumor cells [45]. Liu’s research team [46] has published findings on the application of water-soluble graphene quantum dots. Through steric modification of water-soluble functional groups, researchers achieved precise atomic-level synthesis of quantum dots. These quantum dots exhibited a dark red emission and demonstrated exceptional sensitization and have the potential for in vivo use as anticancer sonosensitizers. Additionally, it has been reported that N-doped graphene quantum dots (N-GQDs) can generate ROS 3 to 5 times more efficiently than conventional sonosensitizers when exposed to US irradiation. This enhanced capability can more effectively trigger the oxidative stress response in tumor cells and promote their apoptosis [47].

A representative inorganic sonosensitizer indicates that the sonochemical catalytic efficiency of N-GQDs is 3 to 5 times higher than that of traditional sonocatalysts (e.g., hematoporphyrin: Ph, manganese porphyrin Mn-Ph, zinc porphyrin Zn-Ph, and TiO_2_). Meanwhile, the sonochemical catalytic efficiency of N-GQDs is much higher than that of the control samples [e.g., amino-modified graphene quantum dots (NH_2_-GQDs), C_3_N quantum dots (C_3_N QDs), and intrinsic graphene quantum dots (GQDs)]. The results indicate that the presence of lattice N atoms in N-GQDs plays a key role in its high sonochemical catalytic efficiency. In order to gain further insight into the catalytic center and mechanism, the authors conducted theoretical calculations and model simulations. The results show that the strong interaction between the pyrrole N and pyridine N in Sp^2^ carbon skeleton is helpful to the catalytic cracking of electron-hole pairs of N-GQDs and water molecules in excited state, thereby efficiently producing active substances [47].

**Fig. 7.**
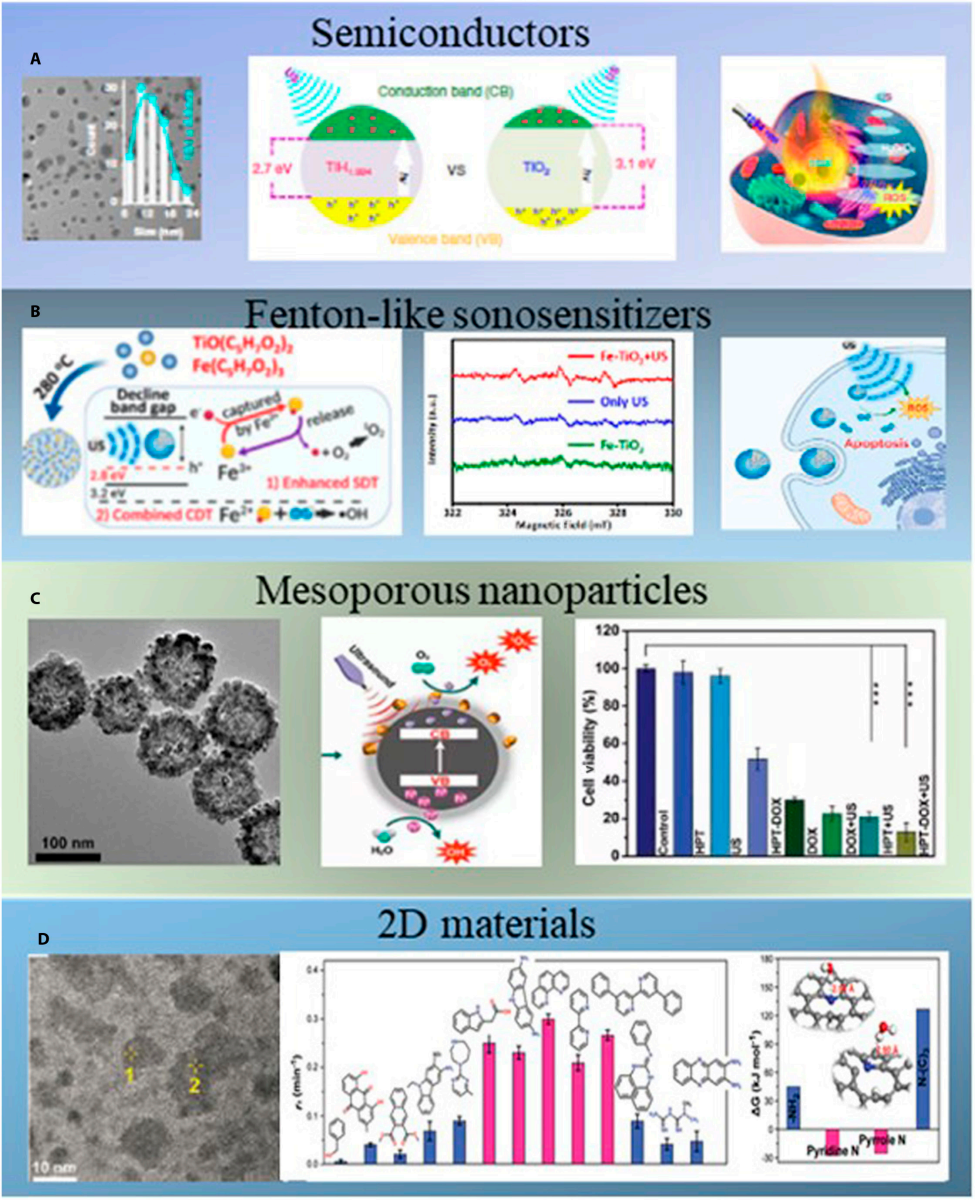
(A) Transmission electron microscopy image and schematic illustration of sonodynamic and photothermal properties of TiH_1.924_ nanodots. Reproduced with permission [34]. Copyright 2020, Nature. (B) Fe-TiO_2_ nanorod’s SDT mechanism. Reproduced with permission [35]. Copyright 2020, American Chemical Society. (C) Schematic illustration of mesoporous Pt-TiO_2_ as enhanced sonosensitizer for sonodynamic cancer therapy. Reproduced with permission [43]. Copyright 2020, Wiley-VCH. (D) Graphene quantum dots show good sonosensitivity. Reproduced with permission [47]. Copyright 2021, Wiley-VCH.

In recent years, there has been a growing body of research on inorganic sonosensitive agents. Researchers use various strategies to design a variety of sonosensitizers to improve the response of nanomaterials to US energy (Fig. 7), with the goal of generating increased free radicals and eradicating tumor cells. This opens up infinite possibilities for the design of sonosensitive agents. However, the mechanisms of inorganic nanoparticles in suppressing the cavitation threshold in SDT need further research.

**Fig. 8.**
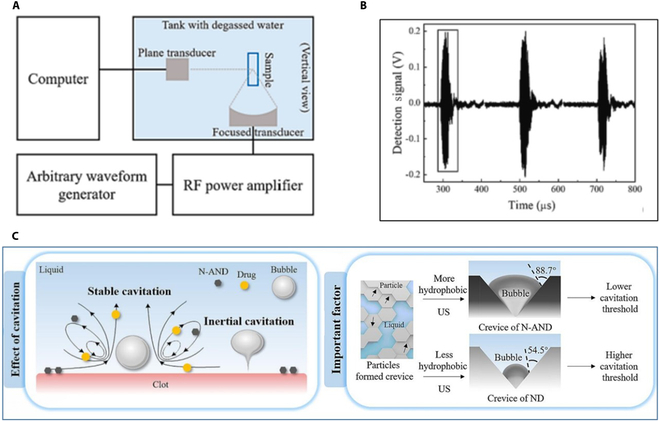
(A) Setup of passive cavitation detection experiments. (B) Detected cavitation signal from inertial cavitation. (C) Schematic diagram of SDT promoted by nitrogen-doped nanodiamonds (modified from [49]). Copyright 2023, Elsevier.

A new type of carbon nanomaterial has been applied to US thrombolysis. Nanodiamonds (NDs) are receiving increasing attention as an ideal tool for disease diagnosis and drug delivery due to their unique modifiability, low cytotoxicity, good chemical stability, and biocompatibility [48]. A nitrogen-modified ND exhibits enormous potential in assisting US thrombolysis due to its high cavitation ability. Researchers used hydrophones to detect acoustic cavitation models triggered by NDs, and the signal data were analyzed (Fig. 8A and B). Cavitation leads to mixing and flow effects, enhancing the efficacy of thrombolytic drugs. On the other hand, the violent oscillation of bubbles also significantly improves the thrombolysis effect (Fig. 8C). The author attributes the cavitation ability of nitrogen-doped annealed nanodiamond (N-AND) to its microstructure and surface properties. In particular, the presence of gaps in particle clusters provides favorable conditions for the formation of cavitation nuclei and reduces the cavitation threshold (Fig. 8C) [49].

In fact, the TME and the complexities of SDT process present challenges to the effectiveness of treatment. To address this, researchers have explored strategies such as introducing defect-repaired carbon nitride nanomaterials [50] and utilizing Z-scheme heterojunctions sonosensitizers [51] to enhance electron-hole separation and sensitize semiconductors for improved SDT outcomes. Overall, the development of innovative sonosensitizers and nanoplatforms has shown great promise in augmenting SDT and overcoming limitations associated with traditional approaches. By strategically designing high-efficiency sonosensitizer-derived theranostics, researchers aim to accelerate the clinical translation of SDT for various therapeutic applications.

#### Nanoparticles derived from a metallo-organic precursors

Nanoparticles derived from a metallo-organic precursor provide a potential alternative method for the preparation of inorganic sonosensitizers. By subjecting these precursors to high-temperature pyrolysis, they can be conveniently transformed into conventional nanostructured inorganic functional materials. It should be noted that the precursor’s organic ligands are transformed into porous graphitic carbon, while the metal ions are often reduced to nanoscale metal or metal oxide particles. This differs from metal–organic complexes. Liu’s research team [52] made the groundbreaking discovery that mesoporous carbon spheres with a porphyrin-like structure, derived from a metal–organic framework (MOF), can serve as highly effective inorganic sonosensitizers for SDT. They successfully addressed the limitations associated with traditional organic sonosensitizers, such as low water solubility, rapid clearance from the bloodstream, and limited accumulation at tumor sites. Lin and colleagues [43] constructed a titanium-based metal–organic skeleton [D-MOF (Ti)] enriched with defect sites that exhibited significant sonosensitivity and used it to enhance SDT. Compared with the commonly used TiO_2_ sonosensitizers, D-MOF (Ti) generated higher ROS yield under US stimulation because of its narrower energy bandgap. Meanwhile, the Ti^3+^ present in D-MOF (Ti) showed a high level of Fenton-like reactivity, leading to significant synergy in treating tumors.

Moreover, researchers have synthesized double-layer hollow manganese silicate nanoparticles (DHMs) from a metallo-organic precursor. These DHMs can efficiently generate ROS under US irradiation and serve as guides for SDT through multimodal imaging. The presence of Mn in DHMs not only improves the separation of electrons and holes but also increases the production of ROS [53]. It has the capability to catalyze the conversion of hydrogen peroxide into oxygen within the TME, thereby modifying the tumor’s hypoxic conditions and enhancing its antitumor effects. In vivo studies confirmed that DHMs, guided by US and magnetic resonance imaging (MRI), effectively inhibited tumor growth through SDT. This research implies that nanosized particles originating from a MOF can provide both sonosensitivity and oxygen production capabilities, thereby offering substantial therapeutic potential for hypoxic tumors. Hang et al. [54] synthesized Mn (II)-chelated Gd-TCPP nanosheets for MRI-guided SDT and chemodynamic therapy, showcasing the potential of multifunctional sonosensitizers.

Generally, some organic sonosensitizers have been approved for clinical use, but their stability is not good. Alternatively, inorganic materials could produce relatively more ROSs, while the clinical application prospects of inorganic materials are often criticized. However, their structure is relatively simple, allowing researchers to gain a glimpse into the mechanism by which sonosensitizers respond to US stimulation and generate free radicals. However, due to the location of cavitation bubbles being random, it is difficult to quantify the energy of US stimulation on sonosensitive agents. This brings a lot of uncertainty and nonrepeatability to the experiment of sonosensitizers. Thus, we propose that building a controllable US/sonosensitizer system may require combining the controllability of MBs with the advantages of material sonosensitizers that can generate sufficient ROS.

## Metal–Organic Hybrid Systems

Researchers have realized the problem of insufficient stability of organic sonosensitive agents, and therefore adopt an inorganic–organic hybrid strategy to improve the stability and effectiveness of sonosensitizers. Certain metal–organic hybrid systems have also shown good sonosensitivity (Table 3). Huang et al. have developed a nanoscale platform based on a MOF (Zr-MOF@AIPH), which can generate singlet oxygen and oxygen-independent alkyl radicals under US stimulation. This metal–organic sonosensitizer enhances the therapeutic effect of SDT in hypoxic tumors [55]. An et al. [56] have reported that [Ru (bpy)_3_]^2+^ can be excited under US irradiation. When oxygen is present, US-induced excitation of [Ru (bpy)_3_]^2+^ enables the transfer of energy to oxygen, resulting in the generation of singlet oxygen radicals. This process facilitates effective SDT for tumor treatment. This significant advancement has opened up possibilities for utilizing a variety of metal–organic complexes in noninvasive US-based cancer therapy. The combination of the sonodynamic drug HMME with Fe ions can produce an Fe-HMME complex. The HMME can produce singlet oxygen in response to US, while the Fe ions allow MRI. The porosity and negative charge of the nanoparticles can be tailored to enable efficient loading of the anticancer drug doxorubicin. Its release can be initiated by changes in pH and US. This combination has demonstrated favorable therapeutic outcomes in treating tumors in the deep body, offering fresh insights for the development of alternative metal–organic nanoscale sonosensitizers [57]. Chen and colleagues [58] have created ultrasmall Gd^3 +^-hematoporphyrin nanoparticles capable of tissue penetration and ROS production when exposed to US. In vivo studies have shown their effectiveness in SDT. Typically, the metal–organic combination provides excellent stability for organic sonosensitizers, resulting in increased ROS production for tumor therapy.

**Table 3.** Metal–organic hybrid systems for SDT

Sonosensitizers	Application	US conditions	Reference
Hemoglobin@metal–organic frameworks	Breast cancer cells	1 MHz, 1.5 W cm^−2^, 50% duty cycle, 1 min	[89]
Iridic–porphyrin complex	Breast cancer cells	3.0 MHz, 1.5 W cm^−2^, 0.3 min	[90]
Pro-oxidant drug-loaded porphyrinic zirconium metal–organic frameworks	Breast cancer cells	1 MHz, 0.3 W cm^−2^, 2 min	[91]
Tetra-a-(3-carboxyphenoxyl) zinc(II) phthalocyanine conjugated with bovine serum albumin	Hepatocellular carcinoma cells	1 MHz, 2.0 W cm^−2^, 3 min	[92]
Polyion complex micelles incorporating TiO_2_ nanoparticles	Cervical carcinoma cells	1 MHz, 5 W cm^−2^, 10% duty cycle, 2 min	[93]
Polypyrrole-coated mesoporous TiO_2_ nanocomposites	Hepatoma cells	1 MHz, 1.5 W cm^−2^, 60 s	[94]
Poly-amino acid nanoparticles bearing Fe^3+^	Breast cancer cells	1 MHz, 2.5 W cm^−2^, 50% duty cycle, 5 min	[95]
Mn-methylphenylporphyrin Zn-methylphenylporphyrin TiO-methylphenylporphyrin	Breast cancer cells	1 MHz, 2 W cm^−2^, 50% duty cycle, 3 min	[59]
Cubic porphyrin-based covalent organic framework	Breast cancer cells	1 MHz, 1.5 W cm^−2^, 50% duty cycle, 10 min	[96]
Covalent organic framework–TiO_2_ nanocomposite	Breast cancer cells	1 MHz, 1.5 W cm^−2^, 5 min	[97]
Erythrocyte-camouflaged mesoporous TiO_2_ nanoplatform loaded with a hypoxia-activated drug	Breast cancer cells	1 MHz, 1 W cm^−2^, 60 s	[98]
Copper–cysteamine	Breast cancer cells	1 MHz, 2 W, 3 min	[99]
An alkyl radical generator on a zirconium metal–organic framework	Pancreatic cancer cells	1 MHz, 1 W cm^−2^, 1 min	[55]
Erythrocyte membrane-camouflaged nanocarriers integrated with platinum nanoparticles and glucose oxidase	Pancreatic cancer cells	3 MHz, 0.1 W cm^−2^, 5 min	[100]
Fe-hematoporphyrin	Breast cancer cells	1.75 W cm^−2^, 10 min	[57]
Hollow ferro and ferric oxide particles bearing hematoporphyrin	Prostate cancer cells	3 MHz, 0.1 W cm^−2^, 5 min	[101]
Rh/sparfloxacin/human serum albumin	Fibroblast-like synoviocyte	1 MHz, 1 W cm^−2^, 1 min	[102]

**Fig. 9.**
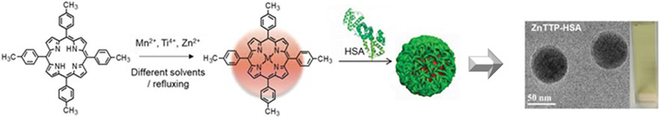
4-Methylphenylporphyrin (TPP) was used to coordinate with Mn^2+^, Zn^2+^, and Ti^4+^ to synthesize MTPP complexes. Reproduced with permission [59]. Copyright 2018, Wiley-VCH.

Porphyrin and its derivatives are the most widely used organic sonosensitizers in SDT. However, these porphyrins often suffer from issues related to poor chemical and biological stability, necessitating further optimization. A recent work has revealed that metalloporphyrin complexes and their nanoparticles offer a means to overcome these obstacles. Cai’s team [59] used 4-methylphenylporphyrin (TPP) to coordinate with Mn^2+^, Zn^2+^, and Ti^4+^ to synthesize 3 kinds of organic complexes (Fig. 9). Then, human serum albumin (HSA) was added to form metal 4-methylphenylporphyrin (MTPP)-HSAs, enhancing its solubility in water. The dispersion of MTPP-HSAs in fetal bovine serum was measured by ultraviolet-visible absorption spectrophotometer. The obtained sonosensitizer exhibits a smooth spherical structure. Notably, experimental findings demonstrated that, under similar conditions, MnTTP is more easily excited than other complexes and can produce more singlet oxygen. This finding highlights the potential of metalloporphyrin complexes as superior organic sonosensitizers for SDT applications, demonstrating the ability of metal–organic hybrid systems in the field of SDT.

## Cellular Sonosensitizer

In recent years, cell surface engineering has attracted increasing attention as a new research field. More and more research is dedicated to explore the impact of cell material modification on cell properties, opening up its application prospects in energy storage, photosynthesis, biocatalysis, and other fields. In the field of SDT, there have also been reports of using cells as novel sonosensitizers. These cellular sonosensitizers combine the advantages of bubble contrast agents and material sonosensitizers, making them a new research direction worthy of exploring.

In a report using live neutrophils as cellular sonosensitizers, researchers encapsulated oxygen-containing PFCs and temorphin into peptide-modified multilayer liposomes (C-ML/HPT/O_2_) and then loaded the liposomes into live neutrophils to prepare Acouscyte/O_2_. Acouscyte/O_2_ not only carries a large amount of oxygen but also has the characteristics of long-term internal circulation, recruitment, and decomposition triggered by inflammation. Studies have shown that Acouscyte/O_2_ can selectively accumulate in tumors, effectively increase oxygen levels within the tumor, trigger sonodynamic anticancer therapy under US stimulation, and achieve the goal of tumor ablation. This strategy can effectively prolong the survival time of tumor-bearing mice with minimized side effects. Therefore, the Acouscyte/O_2_ platform can not only efficiently remove tumors but also monitor tumors in real time to realize visual diagnosis and treatment [60].

**Fig. 10.**
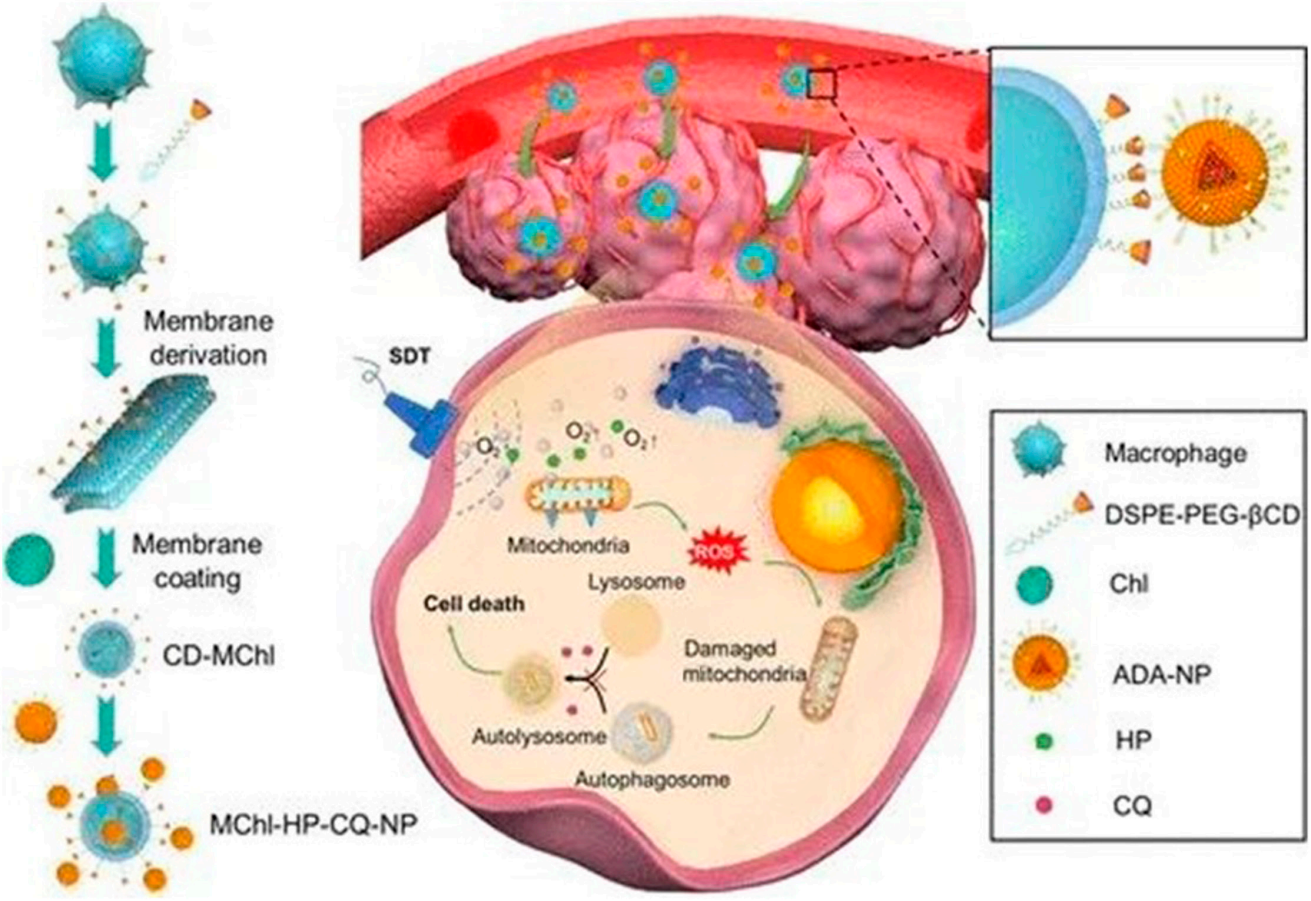
The macrophage membrane was coated on *Chlorella* cells to increase the accumulation of green algae cells in tumors [63]. Copyright 2023, American Chemical Society.

Microalgae are a kind of natural single-cell microorganisms with photosynthetic ability. They are widely used in fields of biofuels, food, and health products. New progress has also been made in the in vivo cancer treatment with the help of engineering active microalgae [61]. Live algae stand out in the field of medicine due to its inherent unique advantages, including its contents with strong biological functions, the ability to improve oxygen and ROS production under light, the consumption of glucose at wound areas, autonomous chemotaxis and blood clot penetration, high editability, low cost, and good biosafety [62]. However, microalgae, as an exogenous microorganism, may cause immune response after intravenous injection and can be removed before aggregation in tumor tissue. Therefore, improving the biocompatibility and drug delivery efficacy into tumor sites is one of the challenges faced by microalgae in vivo. In the field of SDT, Wang and colleagues [63] constructed macrophage membrane-coated *Chlorella* cells (MChl) to increase the accumulation of green algae cells in tumors (Fig. 10). To inhibit autophagy protection, β-cyclodextrin (β-CD) was used to modified MChl (CD MChl). In addition, through the host–guest interaction of adamantane (ADA)-modified liposomes (ADA-NP), MChl is conjugated with drug-loaded liposomes to form MChl-NP. This strategy can promote target delivery of drug-loaded liposomes to melanoma and then enhance the efficacy of SDT by alleviating hypoxia and inhibiting autophagy. In addition, living *Chlorella* can sustainably alleviate tumor hypoxia-related characteristics and reverse the immunosuppressive microenvironment through continuous oxygen production. The synergistic therapy achieved by local oxygen production, SDT, and autophagy inhibition has greatly improved the therapeutic effect of this cellular sonosensitizer on melanoma.

During the development of different types of cellular sonosensitizers, Xu and colleagues [64] proposed a nanocarrier composed of macrophage membrane-modified nanocarriers, encapsulating copper-doped zeolite imidazolium salt framework-8 (Cu@ZIF-8). Thus, a cell-derived intelligent nanorobot (SonoCu) loaded with PFC and organic sonosensitizer Ce6 was used to synergistically trigger SDT via copper-induced cell death. SonoCu can not only improve its accumulation at tumor sites and uptake efficiency by cancer cells through cell membrane camouflage but also enhance tumor blood flow and oxygen supply in response to US stimulation to overcome treatment barriers and activate sonodynamic copper death. This study has found that the effectiveness of SDT can be further amplified by a variety of cell death mechanisms medicated by copper, including accumulation of ROS, protein toxicity stress, and metabolic regulation, which can synergistically cause cancer cell death.

In addition to utilizing cell surface modification engineering to achieve immune escape, drug loading, sonosensitivity, and other functions, genetic engineering has also been used to modify cells to prepare cellular sonosensitizers. It has been reported that genetically engineered bacteria can produce submicrometer bubbles in the cytoplasm through heterologous expression of gene clusters, which can significantly enhance the sonosensitivity of engineered bacteria GVs@*E.coli*. The improvement of sonosensitivity will allow engineered bacteria to be manipulated by US pulses. As a result, GVs@*E.coli* in the circulatory system can be programmatically controlled as needed. In addition, the use of acoustic tweezers can significantly promote the migration and retention of engineered cells to the tumor site, and effectively slow down the tumor growth [65]. The in vivo acoustic manipulation technology based on the combination of biosynthetic gas vacuoles and acoustic tweezers provides a targeted driving method for cell therapy in biomedical applications. Another team also used US to control the expression of interferon by bacteria in tumors [interferon-γ (IFN-γ)]. It has successfully realized the immunotherapeutic application on tumors [66].

Early nanosensitizers mainly used rigid metal and polymer structures, allowing for various in vitro applications. Cellular sonosensitizers provide some unique advantages for in vivo operations, such as improved drug delivery efficiency and deep tissue imaging ability. Chemically or genetically modified cells can exhibit good acoustic responsiveness to improve the SDT effects. This is a highly anticipated development direction.

## Conclusions and Future Perspectives

US is a safe, noninvasive, and convenient energy carrier, with small energy loss when passing through different media. Currently, the application and manipulation of bioeffects induced by US mechanical effects (e.g., cavitation, microfluidics, scattering, and acoustic radiation) have attracted broad attentions in the interdisciplinary fields of physics, medicine, and bioengineering.

The typical SDT refers to the use of US to stimulate sonosensitizers, which can effectively activate cavitation activity, promote ROS production, and facilitate the extinction of harmful cells (e.g., tumor cells and bacteria). As reviewed in this article, in order to improve the ability of acoustic sensitizers to produce ROS, various types of sonosensitizers have been developed, such as bubbles, organic sonosensitizers, inorganic materials, organic–inorganic hybrids, and cellular sonosensitizer.

When MBs possess the characteristics of US imaging ability and US response performance, they can have the advantages of visualization and precise drug release control, which will also help develop related integrated platforms for disease diagnosis and treatment. This type of sonosensitizers is easy to modify. Efforts are being made for the approval of clinical use. However, the impact of artificially modified shell structures on the acoustic properties of MBs remains to be explored. The main issue with MBs or nanobubbles is that an overly stable shell structure may weaken the acoustic response performance of the MBs, thereby limiting US-controlled delivery of drugs.

The use of diagnostic and therapeutic US to activate cavitation and produce free radicals by stimulating organic/inorganic sonosensitizers is a hot topic in SDT research. Organic sonosensitizers exhibit excellent sonodynamic effects, while inorganic sonosensitizers have stable chemical structures, and the generation rate of their ROS is controllable. However, the stability, biocompatibility, and/or sonosensitivity of these sonosensitizers are relatively low, which greatly limits the development of SDT. Alternatively, inorganic nanomaterials can provide cavitation nucleation sites, lower cavitation thresholds, and facilitate energy transfer, which enhances ultrasonic chemical, thermal, and biological effects. However, the precise mechanisms underlying the sonodynamic phenomenon still require further investigation. But these materials often have not been approved for clinical application. From the perspective of advanced SDT technology, a relatively simple composition of sonosensitizers with a metal–organic hybrid system combining the advantages of organic photosensitizers and inorganic sonosensitizers can help researchers gain deeper understanding of the mechanism of free radicals produced by SDT. It should be noted that there are still many concerns in the clinical application of nanomaterials.

On the other hand, cell surface modification engineering is used to prepare cellular sonosensitizers that can achieve the functions of immune escape, drug loading, gas carrying, sonosensitivity, and so on. Natural cells have a uniform size, which is an advantage for them as bubble-type sonosensitizers. However, researchers are increasingly pursuing nanosized sonosensitizers for better acoustic signals. In this context, how micrometer-sized cell-type sonosensitizers can highlight their indispensable advantages is yet to be explored. Additionally, there are few reports on the acoustic properties of cell-type sonosensitizers. However, further exploration is needed on the acoustic properties, ability of ROS production, and the potential clinical application of cellular sonosensitizer.

In general, the remarkable advantage of US lies in its ability to provide a noninvasive and nonradiation treatment option for tumors deep in the body. In the past years, based on decent assessment of ROS production, practical treatment efficiency, and potential adverse reactions, researchers have made great efforts to improving the acoustic and chemical properties of sonosensitizers so that more effective SDT could be achieved. However, how to accurately quantify the energy provided by US radiation to sonosensitizers remains a challenge. The advantage of bubble- or cellular-type sonosensitizers is that they can treat MBs or cells as cavitation bubbles, detect and analyze the energy of cavitation bubbles through various technologies, and correlate the corresponding ROS production, nanoparticle transport efficiency, and mechanical and biological effects for mechanism investigations. From the perspective of implementing clinical applications, future research should give priority to the realization of precise manipulation and optimization of SDT-mediated therapy while conducting in-depth investigations on the mechanisms underlying US-mediated SDT effect.

Although sonosensitizers offer significant potential in enhancing cancer treatment through SDT, a thorough evaluation of their long-term safety and potential side effects remains essential. Unfortunately, current research on this aspect is limited. Considering the long-term safety and potential side effects of sonosensitizers is imperative in the development and implementation of SDTs. Detailed investigations into toxicity profiles, pharmacokinetics, immunogenic responses, and localized effects will help ensure a comprehensive understanding of these agents. Ultimately, the establishment of thorough follow-up protocols and posttreatment monitoring can contribute to maximizing the therapeutic benefits of sonosensitizers while minimizing risks. Continued research in this area will be essential as we strive to improve cancer treatment outcomes and enhance patient safety.

The concept of personalized medicine focuses on customizing medical treatment to align with the unique characteristics of individual patients, including their genetic profiles, disease characteristics, and personal preferences. For SDT, this approach can maximize therapeutic efficacy and minimize adverse effects. Strategies to tailor SDT treatment plans should be based on specific patient conditions. With regard to the advanced preparation concepts of sonosensitizers discussed in this review, the relevant SDT is still some distance away from clinical trials. We expect that personalizing SDT requires a multifaceted approach that encompasses patient stratification based on tumor biology, customization of treatment agents, optimization of dosing and scheduling, continuous monitoring of treatment responses, and patient-centric practices. Generally, based on the patient’s condition, doctors can collaborate with technicians to customize sonosensitizers loaded with targeted drugs. These sonosensitizers should exhibit specificity toward the lesions and can be eliminated from the body after the treatment process is completed.

As the field of sonosensitizers continues to evolve, continued exploration of new materials, including organic and inorganic nanomaterials, is essential. Research should focus on synthesizing sonosensitizers that exhibit enhanced sonodynamic effects, high stability, and biocompatibility. Investigating biodegradable and environmentally friendly options could align with the increasing emphasis on sustainable practices in medicine. Exploring the synergistic effects of combining sonosensitizers with other therapeutic modalities, such as chemotherapy, radiotherapy, or immunotherapy, could lead to improved treatment outcomes. Future studies should investigate the optimal combinations and timing of these therapies to maximize their therapeutic potential. Conducting more comprehensive in vivo studies and advancing toward clinical trials will be crucial for translating laboratory findings into clinical practice. Addressing challenges related to the bioavailability, pharmacokinetics, and safety profiles of sonosensitizers in human patients will be key steps in this process. A deeper understanding of the underlying mechanisms by which sonosensitizers induce cytotoxicity and activate immune responses could provide insights for optimizing treatment protocols. Advanced imaging techniques and molecular biology approaches should be employed to study these mechanisms in real time and at the cellular level. We expect that collaboration between material scientists, biologists, and clinicians can foster innovations in sonosensitizers. Integrating knowledge from various fields may lead to novel approaches and solutions that can address current limitations in SDT.
